# The Cytotoxic Effect of *Apis mellifera* Venom with a Synergistic Potential of Its Two Main Components—Melittin and PLA2—On Colon Cancer HCT116 Cell Lines

**DOI:** 10.3390/molecules26082264

**Published:** 2021-04-14

**Authors:** Carole Yaacoub, Mariam Rifi, Dany El-Obeid, Hiba Mawlawi, Jean-Marc Sabatier, Bruno Coutard, Ziad Fajloun

**Affiliations:** 1 Laboratory of Applied Biotechnology (LBA3B), Azm Center for Research in Biotechnology and Its Applications, EDST, Lebanese University, Tripoli 1300, Lebanon; carole.yaacoub@etu.univ-amu.fr (C.Y.); maryam.rifi8@gmail.com (M.R.); hiba.mawlawi@ul.edu.lb (H.M.); 2 Unité des Virus Emergents, Aix-Marseille University-IRD 190-Inserm 1207, IHU Méditerranée Infection, 13005 Marseille, France; Bruno.Coutard@univ-amu.fr; 3 Faculty of Agriculture & Veterinary Sciences, Lebanese University, Dekwaneh, Beirut 2832, Lebanon; dany.elobeid@ul.edu.lb; 4 Faculty of Public Health III, Lebanese University, Tripoli 1300, Lebanon; 5 Faculté de Médecine Secteur Nord, 51, Université Aix-Marseille, Institut de NeuroPhysiopathologie, UMR 7051, Boulevard Pierre Dramard-CS80011, CEDEX 15, 13344 Marseille, France; sabatier.jm1@libertysurf.fr or; 6 Faculty of Sciences III, Department of Biology, Michel Slayman Tripoli Campus, Lebanese University, Ras Maska 1352, Lebanon

**Keywords:** *Apis mellifera*, bee venom, melittin, PLA2, in vitro anticancer effect, HCT116 cell lines

## Abstract

Colon carcinogenesis is ranked second globally among human diseases after cardiovascular failures. Bee venom (BV) has been shown to possess in vitro anticancer effects against several types of cancer cells. The two main biopeptides of *Apis mellifera* BV, namely, melittin (MEL) and phospholipase A2 (PLA2), are suspected to be the biomolecules responsible for the anticancer activity. The present work aims to evaluate the cytotoxic effect of the *A. mellifera* venom on human colon carcinoma cells (HCT116), and to assess the synergistic effect of MEL and PLA2 on these cells. After analyzing, through high-pressure liquid chromatography, the proportions of MEL and PLA2 on BV, we have established a cell viability assay to evaluate the effect of BV, MEL, PLA2, and a mixture of MEL and PLA2 on the HCT116 cells. Results obtained showed a strong cytotoxicity effect induced by the *A. mellifera* venom and to a lower extent MEL or PLA2 alone. Remarkably, when MEL and PLA2 were added together, their cytotoxic effect was greatly improved, suggesting a synergistic activity on HCT116 cells. These findings confirm the cytotoxic effect of the *A. mellifera* venom and highlight the presence of synergistic potential activities between MEL and PLA2, possibly inducing membrane disruption of HCT116 cancer cells. Altogether, these results could serve as a basis for the development of new anticancer treatments.

## 1. Introduction

To this date, colon cancer is among the most common human diseases. It is ranked second globally after cardiovascular failures [1] and is considered the third most frequently occurring cancer for both men and women [2]. The current treatments used to treat this type of cancer include chemotherapy, radiotherapy, and surgery—all of which do not yield satisfying results and possess many side effects [2]. Therefore, the development of innovative therapeutics is, therefore, highly needed.

Scientists have been increasingly interested in natural products as an invaluable source of bioactive compounds with limited side effects. Many experiments have demonstrated the antitumor activities of natural extracts such as several venoms/toxins derived from scorpions, snakes, bees, and other venomous animals [3]. Bee venom (BV) is one of the most natural extracts studied in recent years due to its richness in bioactive molecules such as melittin (MEL) and phospholipase A2 (PLA2), which possess a wide range of biological activities that can serve as a basis for the development of new drugs and have a significant positive impact on human health [4]. Traditionally used as an analgesic, BV has been also used in the treatment of chronic inflammatory diseases such as rheumatoid arthritis and multiple sclerosis [5]. Recently, several studies have shown the capacity of BV to induce apoptosis, necrosis, as well as cytotoxicity and growth inhibition of different types of cancer cells [6,7].

MEL is the major component of BV. It has been shown to exert both hemolytic activity and antibacterial activities by inducing pores, fusion, and vesicles in the cell membranes [8]. It can lead to hormone secretion, change of membrane potential, and aggregation of membrane proteins. Additionally, MEL can also stimulate many enzymes such as the protein Kinase C, the cellular PLA2, and the adenylate cyclase [9]. In addition, BV contains PLA2, a well-characterized enzyme that hydrolyzes the fatty acid from the sn-2 position of the phospholipids membrane. As a consequence, fatty acids -particular arachidonic acid- and lysophospholipid are released [10]. Furthermore, PLA2 has antibacterial effects and activates the immune system by stimulating type 2 immune response [11,12,13]. In terms of anticancer activities, MEL has proved effective against different types of cancer cells such as ovarian cancer cells via the activation of death receptors and inhibition of JAK2/STAT3 pathway [14]. Additionally, it can inhibit the growth of human hepatoma and glioma cell lines by inducing their apoptosis [15]. The cooperation between PLA2 and phosphatidylinositol-(3,4)-bisphosphate leads to cell death in the renal cancer cells by destabilizing the membrane [16]. Moreover, the interaction between MEL and PLA2 on cell membranes has been already studied and has shown that the two proteins work synergistically to disrupt the membrane organizations and to act on the F_1_F_0_-ATPase enzyme of the *Escherichia coli* membrane [17,18]. Otherwise, Cajal and Jain have previously validated the ability of MEL to activate PLA2 in different types of vesicles, which might explain the mechanism of action of PLA2 and MEL in disrupting the biological membrane and the existence of a synergistic effect between these two biopeptides [19]

This study aims to evaluate the cytotoxic effects of *A. mellifera* venom and its two main compounds—MEL and PLA2—on human colon cancer cells. It also assesses the effect of these two biomolecules when combined together by studying their cytotoxic effects on HCT116 cell lines.

## 2. Results

### 2.1. Analysis of A. mellifera syriaca Venom (Used in Our Experiments) and Its Two Main Components, MEL and PLA2, by HPLC

**Figure 1:**
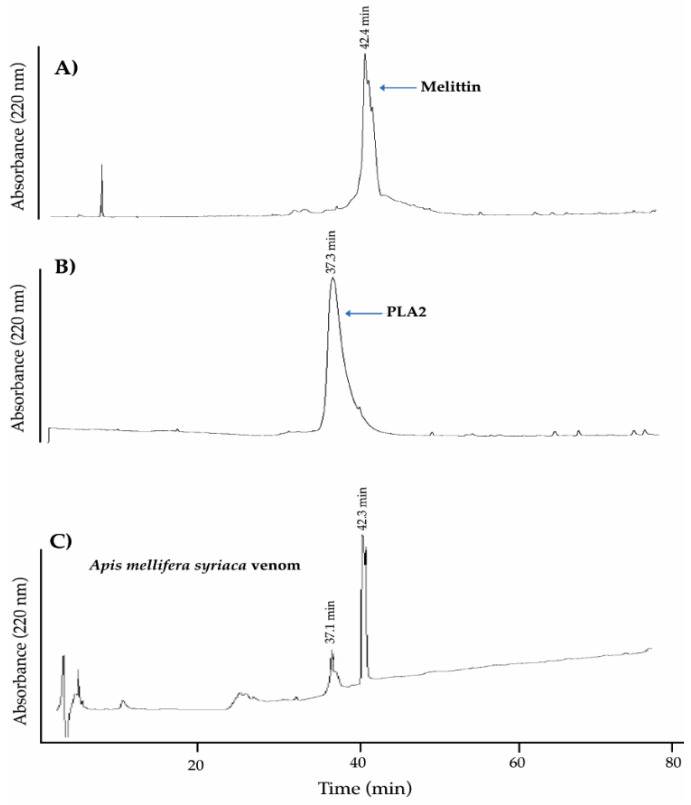
High-pressure liquid chromatography (HPLC) chromatograms showing reverse-phase C18 fractionation of (**A**) standard MEL, (**B**) standard PLA2, and (**C**) *A. mellifera syriaca* crude venom. The two standard molecules MEL and PLA2 were eluted individually at the same retention times as those presented in the *A. mellifera syriaca* venom, validating their conformational properties.

To show that *A. mellifera syriaca* venom contains the same two standard molecules MEL and PLA2 used during our experiments, we used the high-pressure liquid chromatography (HPLC) technique to analyze the different components of this venom focusing on the two main compounds of interests, MEL and PLA2. Figure 1A shows the chromatogram referred to as standard MEL. It reveals a unique peak at a retention time of 42.4 min (t = 42.4 min). This indicates the purity of this molecule, which can be used as a reference for the detection of MEL in *A. mellifera syriaca* venom. Similarly, the chromatogram profile corresponding to the standard PLA2 (Figure 1B) showed a unique peak with a retention time of (t = 37.3 min). However, the chromatogram of *A. mellifera syriaca* crude venom revealed two major peaks (Figure 1C). The first has a retention time of 37.1 min, while the second has a retention time of 42.3 min with the highest intensity. The second clearly corresponds to MEL since it is the most abundant peptide in BV with a percentage ranging between 40% and 60% of its composition [20]. Comparing the results with those of standards MEL and PLA2, we can validate that the second peak in Figure 1C (t = 42.3 min) corresponds to the MEL present in the *A. mellifera syriaca* venom and that the first peak corresponds to PLA2. We calculated the quantity of MEL and PLA2 according to the external standard method, using peak areas and peak heights [21]. According to the results, the *A. mellifera syriaca* venom contains 39.4% of melittin and 11.3% of PLA2, which are in agreement with the literature.

### 2.2. Dose-Dependent Effect of A. mellifera Venom on Cell Viability of Colon Cancer HCT116 Cells

**Figure 2:**
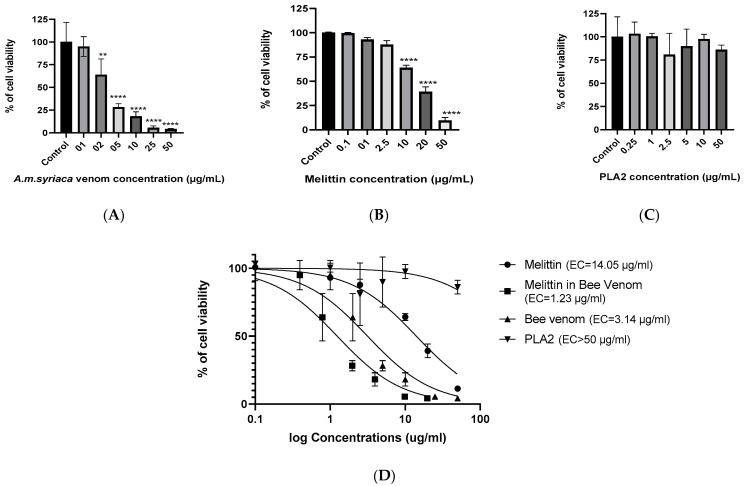
Cell viability of HCT116 colon cancer cells was measured by MTT assay after treatment with increased concentrations of (**A**) *A. mellifera syriaca* venom, (**B**) standard melittin (MEL), and (**C**) standard PLA2. The results represent the mean ± SD of three independent experiments. Statistically significant compared with untreated cells ** *p* < 0.01, and **** *p*< 0.0001. (**D**) Curves for MTT assay showing EC50 values and the % of cell viability in function of log concentrations of standard MEL, MEL in *A. mellifera* venom (titration curve), *A. mellifera* venom (from *A. mellifera syriaca* bees), and standard PLA2. The results represent the mean ±SD of three independent experiments.

The cytotoxic effect of *A. mellifera* venom (from *A. mellifera syriaca* bees) on human colon cancer HCT116 cells was evaluated using MTT (bromure de 3-(4,5-dimethylthiazol-2-yl)-2,5-diphenyl tetrazolium) assay. Cells were exposed to increasing concentrations of BV (1, 2, 5, 10, 25, and 50 µg/mL) for 24 h. The results were expressed as the percentage of cell viability in comparison with the untreated control cells with 100% of viability. The results showed that the BV inhibited the cell viability of HCT116 cells in a dose-dependent manner. In fact, 2 µg/mL of the crude BV was able to induce a significant decrease in cell viability (64% of viability) in comparison to the control. The maximum effect was obtained at a concentration of 50 µg/mL where only 4% of the cells remained viable (Figure 2A). These results reveal the strong cytotoxic effect of *A. mellifera* venom on HCT116 cells with an EC50 of 3.14 µg/mL calculated by Prism software (Figure 2D).

### 2.3. Dose-Dependent Effect of MEL on Cell Viability of Colon Cancer HCT116 Cells

To evaluate the cytotoxicity of MEL on colon cancer HCT116 cells, we tested different concentrations of MEL (0.1, 1, 2, 2.5, 10, 20, and 50 µg/mL). Ten µg/mL of MEL showed significant cytotoxic effect compared to untreated cells with a percentage of 64%. This value further decreased with the increase of MEL’s concentration to reach the lowest percentage of viability (10%) at a concentration of 50 µg/mL (Figure 2B). The EC50 of MEL observed was 14.05 µg/mL (Figure 2D).

### 2.4. Effect of PLA2 on Cell Viability of Colon Cancer HCT116 Cells

To our knowledge, the cytotoxic effect of PLA2 derived from *A. mellifera* venom has never been studied. Yet, the mechanism that we suggested for the anticancer effect of BV is that it is initiated through the activation of PLA2 by MEL. To check the effect of PLA2 from *A. mellifera* venom on cell viability, six concentrations of this enzyme were tested on HCT116 cell lines. The results showed that PLA2 alone do not possess any significant cytotoxic activity within the range of concentrations used in this study (Figure 2C) and that EC50 is more than 50 µg/mL (Figure 2D).

### 2.5. Effect of MEL in A. mellifera Venom on Cell Viability of Colon Cancer HCT116 Cells

To compare the effect of MEL alone and MEL present in the *A. mellifera* venom, we calculated the concentration of the latter through the integration of HPLC peaks concerned. Assuming that in the BV, MEL is the main active molecule responsible of cytotoxicity, we drew a titration curve of cell viability as a function of the log concentration of MEL in BV (Figure 2D). The results highlighted the strong cytotoxic effect of MEL in BV on the human colon cancer cells with EC50 = 1.23 µg/mL. This suggests the presence of specific components in BV that either has an independent cytotoxic effect or promote the cytotoxicity of MEL on colon cancer cells. Among the possible candidates, PLA2 may potentiate MEL’s activity.

### 2.6. Synergistic Effects between MEL and PLA2

**Figure 3:**
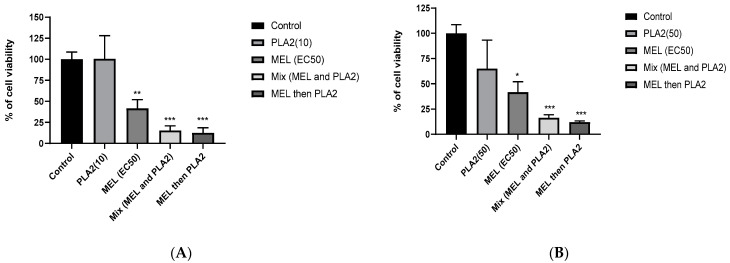
Synergistic effect of MEL and PLA2 on the viability of HCT116 cell lines. HCT116 cells were treated with PLA2 (**A**) (10 µg/mL), or (**B**) (50 µg/mL) and MEL (14.05 µg/mL) simultaneously or after a pretreatment delay. Pretreatment delay corresponds to addition of the MEL 30 min before the PLA2. The data are expressed as the means ± SD (n = 3), * *p* < 0.05, ** *p* < 0.01, *** *p* < 0.001 compared with untreated cells (control).

To study the synergistic effect between MEL and PLA2 -the two main components of *A. mellifera* venom- on human colon cancer cell lines, HCT116 cells were treated with the following: PLA2 alone, MEL alone, PLA2, and MEL, simultaneously or successively with a delay of 30 min. Two concentrations of PLA2 were tested, 10 and 50 µg/mL. As for MEL, a concentration corresponding to its EC50 (14.05 µg/mL) was used. For a concentration of 10 µg/mL of PLA2, the results showed, as expected, that PLA2 had no significant cytotoxic effect when administrated alone (Figure 3A). In contrast, the addition of MEL significantly increased the cytotoxicity of PLA2, compared to the untreated control cells. In fact, when cells were treated with MEL and PLA2 simultaneously, the percentage of cell viability observed was 15.26%, and only 12.5% of cells remained viable when they were pretreated with MEL for 30 min before the addition of PLA2. Finally, to understand more the mechanism of action between MEL and PLA2, we treated the cells with PLA2 and after 30 min we added MEL. The percentage of cell viability obtained was 25%, higher than cells pretreated with MEL, leading us to suggest that MEL facilitates the action of PLA2 on the cell membrane.

Furthermore, PLA2, when added alone at a relatively high concentration (50 µg/mL), was still unable to induce any significant cytotoxic effect on HCT116 cells, while only 42% of the cells remained viable in the presence of MEL alone. When cells were pre-incubated in the presence of PLA2 prior to the addition of MEL, an additional inhibition was observed with a viability percentage of 32% (data not shown). Additionally, when the cells were pre-treated with MEL, the maximum cytotoxicity was observed with a percentage of 12.5% of viable cells. The same effect was obtained with a slight difference when MEL and PLA2 were added together, with a viability percentage of 15.2% (Figure 3B). These results support the above hypothesis that suggests the presence of a synergistic effect between MEL and PLA2.

Finally, the survival rates of HCT116 cells were almost the same in combination of MEL with PLA2 at 10ug/mL and 50 ug/mL, suggesting the synergistic effect was not PLA2-dose dependent, but it depends on the presence of PLA2. This this can be explained by achieving the maximum cytotoxic effect.

## 3. Discussion

In this study, we tested the cytotoxic effects of *A. mellifera* venom obtained from *A. mellifera syriaca* bees, and of its two main components—MEL and PLA2—on human colon cancer HCT116 cells. Our results showed high cytotoxic activity of the BV on the cancerous cell line. Indeed, 2 µg/mL was able to induce a significant cytotoxicity, while 50 µg/mL almost killed the majority of the cells (only 4% of cells remained viable). We also demonstrated a synergistic cytotoxic effect existing between MEL and PLA2 in the venom. The suggestion that the anticancer activity of BV is possibly due to the activation of PLA2 by MEL has been proposed for some time [9]. In addition, it was demonstrated that BV can induce apoptosis by decreasing BCL2 expression and increasing BAX and CASP3 expression in rheumatoid synovial fibroblasts [22]. Moreover, it was found that BV inhibits the growth of different types of cancer cells such as lung cancer line [6], human cervical epidermoid carcinoma CaSki Cells [23], and breast cancer line [24]. It was also able to induce the apoptosis of lung carcinoma cells (NSCLC) by increasing the expression of DR3 expression and inhibition of the NF-κB pathway [25].

Our findings add to the existing literature by showing that MEL has a cytotoxic effect on HCT116 cell lines and acts in a dose-dependent manner. In addition, MEL, as the major component of BV, plays an important role in the cytotoxic effect of the BV. In fact, it was demonstrated that MEL inhibited the ovarian cancer cells via induction of death receptors and inhibition of the JAK2L/STAT3 pathway [14]. It has been also reported that MEL decreased the viability of endothelial progenitor cells and decreased the expression levels of p-AKT and ERK1/2 [26]. Additionally, Liu et al. demonstrated the capacity of MEL to decrease the hepatocellular carcinoma (HCC) metastasis in vivo via the suppression of the Rac1-dependent pathway [27].

When MEL and PLA2 were combined together, a potential cytotoxic synergistic effect was observed and translated by the significant inhibition of the HCT116 cells proliferation. Moreover, the maximum cytotoxic effect was obtained when HCT116 cells were pre-incubated with MEL. In contrast, PLA2 showed no significant cytotoxic effect when added alone. This synergistic effect observed by mixing MEL and PLA2 on HCT116 cells is conformity with the cytotoxic effect observed in the *A. mellifera* crude venom which contains both MEL and PLA2. Additionally, the MEL in the crude venom is much more active than when it is tested alone (see Figure 2D), thus proving the presence of synergy with other molecules in the BV such as PLA2.

Altogether, the results suggested that the synergy of effect between MEL and PLA2 could be due to the potential action of MEL on PLA2, which leads to membrane disruption. We suggest that MEL, like a toxin, could interact and damage the glycocalyx of the cell coat of the cell membrane, thus resulting in free accessibility to the PLA2 to bypass the cell coat and act directly on the phospholipids of the lipid membrane bilayer, which might possibly lead to cell necrosis. Further study must be done to evaluate specific markers related to membrane damage to confirm our hypothesis and to better understand the mechanism of action.

## 4. Materials and Methods

### 4.1. Chemicals and Reagents

The *A. mellifera* venom was collected from the *A. mellifera syriaca* bees, which are found in Matn in Mount Lebanon [18]; the standard MEL and PLA2 were obtained from Latoxan, a French laboratory specializing in animal toxins and poisonous animals; acetonitrile (Acn) was purchased from Scharlau; dimethylsulfoxide (DMSO), culture medium “Dulbecco’s Modified Eagle’s Medium” (DMEM), and MTT kit were purchased from Sigma Aldrich; and HCT116 cells were obtained from the American Type Culture Collection (ATCC).

### 4.2. Analysis of A. mellifera syriaca Venom Components, MEL, and PLA2 by High-Pressure Liquid Chromatography (HPLC) Technique

Chromatographic analysis was carried out using a Discovery^®^ HS C18 25 cm × 4.6 mm, 5 µm column. Ten mg of freeze-dried *A. mellifera* crude venom was dissolved in 1 mL of ultrapure water, and then the solution was filtered using a syringe filter. A volume of 100 µL of the solution corresponding to the maximum volume injected automatically in the HPLC was analyzed. The collection process was done with an elution gradient of 0–60% acetonitrile for 60 min then 60–100% for 20 min at a flow rate of 1 mL/min, and a UV detector at 220 nm to separate the different components of the venom. The elution gradient thus used is composed of two eluents: one polar (eluent A: water) and the other one non-polar (eluent B: acetonitrile). The data were recorded by the HPLC software “HyStar ™”. For MEL and PLA2 standards, 1 mg was dissolved in 1 mL of ultra-pure water. A volume of 20 μL was analyzed. The gradient elution, as well as the time, are the same as HPLC analysis of the *A. mellifera* crude venom. Moreover, the composition of the elution gradient, the flow rate, as well as the wavelengths used are the same.

### 4.3. Cell Culture

HCT116 Cell line, purchased from the American Type Culture Collection, was cultured in DMEM (Gibco Dulbecco’s Modified Eagle Medium obtained from Sigma Eldrish, Beirut-Lebanon) at 37 °C in a humidified atmosphere with 5% CO_2_ and 95% air. Media was amplified with 1% penicillin streptomycin (100 U·mL^−1^) and 10% heat-inactivated fetal bovine serum (FBS).

### 4.4. Cellular Viability Assay

The cellular viability assay was carried out by MTT test. MTT assay relies on the mitochondria of the cell by the enzyme mitochondrial reductase [19]. It converts the yellow dye of MTT to purple formazan. Cells were seeded in 96 well plates at a density of 10^4^ cells/well. At 60–80% confluency, the cells are ready to be treated. Experiments were done in triplicate with different extracts at different concentrations. The treatment was for 24 h. After 24 h, the media was discarded. One hundred µL of MTT solution was added to each well. The absorbance was measured by ELISA READER at 570 nm.

### 4.5. Statistical Analysis

The results were obtained using one-way ANOVA with Bonferroni’s multiple comparison test using the GraphPad Prism software. They were presented as mean ± SD of at least three independent experiments. Statistical significance was defined as * *p* < 0.05, ** *p* < 0.01, and *** *p* < 0.001 compared to untreated cells.

## 5. Conclusions

In this work, we demonstrated that *A. mellifera* venom obtained from *A. mellifera syriaca* bees and MEL induce cell death of HCT116 cells. We demonstrate for the first time the presence of a synergistic cytotoxic effect between both MEL and PLA2 on human colon cancer cells. Our results clearly imply that MEL significantly enhances the activity of PLA2 in human colon cancer cells, which highlights the need for the MEL for the cytotoxic effect of PLA2. Finally, these findings could serve as a basis for the development of new therapeutics to target cancer cell lines, although the specificity of the activity is yet to be addressed.

## Data Availability

Not applicable.

## Sample Availability

Samples of the compounds are not available from the authors.
